# Microbial dynamics and vertical transmission of *Escherichia coli* across consecutive life stages of the black soldier fly (*Hermetia illucens*)

**DOI:** 10.1186/s42523-024-00317-4

**Published:** 2024-05-26

**Authors:** Noor Van Looveren, Freek IJdema, Niels van der Heijden, Mik Van Der Borght, Dries Vandeweyer

**Affiliations:** KU Leuven, Geel Campus, Department of Microbial and Molecular Systems (M2S), Research Group for Insect Production and Processing, Kleinhoefstraat 4, Geel, 2440 Belgium

**Keywords:** *Hermetia illucens*, Bacterial community, Vertical transmission, Foodborne pathogens, 16S rRNA gene sequencing

## Abstract

**Background:**

The black soldier fly (BSF, *Hermetia illucens* L.) is one of the most promising insects for bioconversion of organic waste, which often carry a high microbial load with potential foodborne pathogens. Although horizontal transmission (from rearing substrate to larvae) has been extensively studied, less is known about vertical transmission of microorganisms, and particularly of foodborne pathogens, across different BSF life stages.

**Results:**

This study investigated the microbial dynamics and vertical transmission of *Escherichia coli* across different life stages (larvae, prepupae, pupae and adults) of one BSF life cycle and its associated substrate (chicken feed) and frass, based on a combination of general microbial counts (based on culture-dependent techniques) and the bacterial community composition (based on 16S rRNA gene sequencing). Multiple interactions between the microbiota of the substrate, frass and BSF larvae were affirmed. The larvae showed relative consistency among both the microbial counts and bacterial community composition. Diversification of the bacterial communities started during the pupal stage, while most notable changes of the microbial counts and bacterial community compositions occurred during metamorphosis to adults. Furthermore, vertical transmission of *E. coli* was investigated after substrate inoculation with approximately 7.0 log cfu/g of kanamycin-resistant *E. coli*, and monitoring *E. coli* counts from larval to adult stage. Although the frass still contained substantial levels of *E. coli* (> 4.5 log cfu/g) and *E. coli* was taken up by the larvae, limited vertical transmission of *E. coli* was observed with a decreasing trend until the prepupal stage. *E. coli* counts were below the detection limit (1.0 log cfu/g) for all BSF samples from the end of the pupal stage and the adult stage. Additionally, substrate inoculation of *E. coli* did not have a substantial impact on the bacterial community composition of the substrate, frass or different BSF life stages.

**Conclusions:**

The fluctuating microbial counts and bacterial community composition underscored the dynamic character of the microbiota of BSF life stages. Additionally, vertical transmission throughout one BSF life cycle was not observed for *E. coli*. Hence, these findings paved the way for future case studies on vertical transmission of foodborne pathogens across consecutive BSF life stages or other insect species.

**Supplementary Information:**

The online version contains supplementary material available at 10.1186/s42523-024-00317-4.

## Background

The black soldier fly (BSF, *Hermetia illucens* L., Diptera: Stratiomyidae) is a holometabolous insect which undergoes a complete metamorphosis from larva to adult, with intermediate (pre)pupal stage [1]. The larvae of this insect are gaining increasing interest, as they can grow on an extensive variety of organic waste streams, originating from agriculture, food processing or food waste processing. These substrates can be converted by the larvae into valuable biomass with a favourable nutritional profile. In addition, BSF larvae can be easily mass reared in containers [2, 3]. Consequently, the BSF, and mainly its larvae, emerges as one of the most promising insect species for use as a protein-rich animal feed ingredient [4].

As for other feed ingredients, microbiological safety of BSF larvae should be warranted, especially when using organic waste streams as rearing substrate. Organic waste typically carries a substantial microbial load, potentially including pathogens that pose a risk to humans or animals [5]. Accurate knowledge about the occurrence and behaviour of these foodborne pathogens in organic substrates, as well as potential transmission from the substrate to larvae (horizontal transmission) and across BSF life stages (vertical transmission) is important to gain more insight into the microbiological safety of insects intended for use as feed (and food) [6].

Numerous studies have investigated the horizontal transmission of several food pathogens, such as *Bacillus cereus*, *Escherichia coli*, *Listeria monocytogenes*, *Salmonella* spp. and *Staphylococcus aureus* [5, 7–12]. While these studies uniformly reported the ingestion of the pathogenic species by BSF larvae, the outcomes varied. Some studies demonstrated a reduction, or even complete suppression, of a particular foodborne pathogen [5, 8, 10], while others did not observe a significant decrease in the pathogen levels within the substrate and/or the larvae [7, 9, 12]. These results emphasise the importance of case studies to fill existing knowledge gaps, as horizontal transmission of foodborne pathogens is influenced by several factors, including the substrate, insect species and pathogen species and level [6]. In contrast to an increasing number of studies on horizontal transmission of food pathogens during BSF rearing, studies investigating vertical transmission across BSF life stages or life cycles are, to the best of our knowledge, absent. Filling this knowledge gap is essential to completely understand the pathogen transmission processes associated with BSF.

Additionally, since a few years, it has become increasingly evident that the gut microbial community of the BSF substantially influences its growth, development, health, and defense mechanisms against pathogenic threats [13, 14]. The dynamic nature of this microbial community plays a crucial role in the ability of BSF larvae to digest diverse organic substrates [13, 15–17]. Moreover, the gut microbiota is significantly modulated by different biotic and abiotic factors, such as the rearing substrate, larval age or developmental stage [16, 18–20]. The substrate administered to the larvae represents by far the most studied factor in the context of host-microbiota interactions, and the majority of studies only focused on the larval stage reared on these substrates [19, 21–27]. Additionally, the microbial community of BSF frass, which consists of non-consumed feeding substrate, insect excrements, exuviae and (parts of) dead insects after harvesting the larvae, and can be used as plant fertiliser or soil amendment, has been studied in the last few years [24, 28–30].

Recently, more studies started to explore the influence of the life stages on the microbial community of the BSF [15, 31, 32]. This is elucidating information to understand BSF microbial community dynamics, given that the gut microbiota may have a substantial impact on the digestive processes and maturation of the adult BSF stage [33]. Indeed, metamorphosis of the BSF, a process marked by the remodelling of the gut, is driven by the different digestive requirements between the larval and adult stage. A turnover of the gut microbial community is concomitant with this remodelling process [1, 34]. Nevertheless, further in-depth research on the microbial community of consecutive BSF life stages is recommended.

The objective of this study was dual. Firstly, this study aimed to augment the existing knowledge concerning the microbial dynamics throughout different BSF life stages and its associated substrate and frass. Both culture-dependent and culture-independent (16S rRNA gene sequencing) techniques were used to gain insight in the microbial counts and bacterial community dynamics, respectively, of the substrate and frass, and throughout the larval, prepupal, pupal and adult BSF stage of one rearing cycle. Secondly, an initial case study on the vertical transmission of a single, potentially pathogenic bacterial species, in this case *E. coli* as model organisms, was conducted. Following inoculation of *E. coli* into the rearing substrate of the larvae, *E. coli* counts were monitored in the substrate, the frass and consecutive BSF stages. Additionally, the impact of introduction of *E. coli* on the bacterial community composition of the BSF, the substrate and the frass was examined.

## Materials and methods

### Black soldier fly rearing conditions

Eggs of BSF were obtained from a colony maintained by the Centre of Expertise Sustainable Biomass and Chemistry (Thomas More University of Applied Sciences, Geel, Belgium). A plastic tray containing 1 g of eggs was placed on the nursery substrate, consisting of a mixture of 100 g of chicken starter feed (Chicken Start Mash 259, AVEVE, Belgium) and 100 mL of tap water. The nursery substrate with eggs was incubated in a climate room at 27 °C and 60% Relative Humidity (RH). The egg tray was removed after 4 days, and the nursery substrate with newly hatched larvae was added to an open plastic container (29 cm × 19.5 cm × 18 cm), filled with 240 g of chicken starter feed mixed with 360 mL of tap water. The container was incubated at 27 °C and 60% RH until 8 days after hatching (DAH 8). At DAH 8, a fresh substrate, containing 1100 g of chicken feed and 1300 mL of tap water was prepared in an open plastic container (35 cm × 27 cm × 9.5 cm) and served as substrate for the so-called ‘control cycle’. For monitoring *E. coli* in the BSF life cycle, 50 mL of the tap water was replaced by 50 mL of an *E. coli* suspension (as prepared in ‘*E. coli* strain and inoculation of the substrate’) to prepare the substrate for the so-called ‘inoculated cycle’. To obtain an appropriate larval density, approximately 90 g of the substrate with 8-day-old larvae (one sixth of the entire mass of nursery substrate with larvae), which corresponds to approximately 6000 to 7000 larvae, were added to the (inoculated) substrate. The (inoculated) substrate with larvae was gently mixed with a tablespoon and the open containers containing the control or inoculated cycle were incubated in separate climate chambers (WEISS Pharma 600, Weiss Technik, Liedekerke, Belgium) at 27 °C and 60% RH to avoid cross-contamination. Each 2 days, tap water was visually added to avoid desiccation of the substrate. For the inoculated cycle, the substrate was inoculated with 50 mL of a similar *E. coli* suspension for a second time at DAH 18 to evaluate the impact of older larvae and especially of the following BSF stages which are separated from the substrate (prepupae, pupae and adults) on the presence of *E. coli* inside their body. When prepupae accounted for more than 50% of the population (DAH 23–24), an excess of water (approximately 1000 mL) was added to the substrate, resulting in crawling out of the prepupae. These prepupae were collected in a plastic box (20 cm × 15 cm × 11.5 cm) covered with a lid with small holes to allow air circulation and covered with a mesh to prevent escape of emerged flies. When emergence of the adults had started (DAH 36–38), the box without lid was transferred to a mesh tent (40 cm × 40 cm × 60 cm), together with some water, provided by a plastic box (18 cm × 12 cm × 7 cm) filled with water and covered with paper wipes, and wooden strips for egg deposition, placed onto a box with decaying substrate (consisting of chicken feed and water in a 1:1 ratio (w/w)) to attract the flies by smell, covered with a lid consisting of a mosquito net to make the decaying substrate inaccessible for the flies. The tent was placed in a climate chamber at 27 °C and 60% RH, with day-night cycle of 12 h:12 h, until DAH 46, when the flies started to die. Both the control cycle and inoculated cycle were repeated in triplicate (three biological replicates).

### *E. coli* strain and inoculation of the substrate

**Fig. 1:**
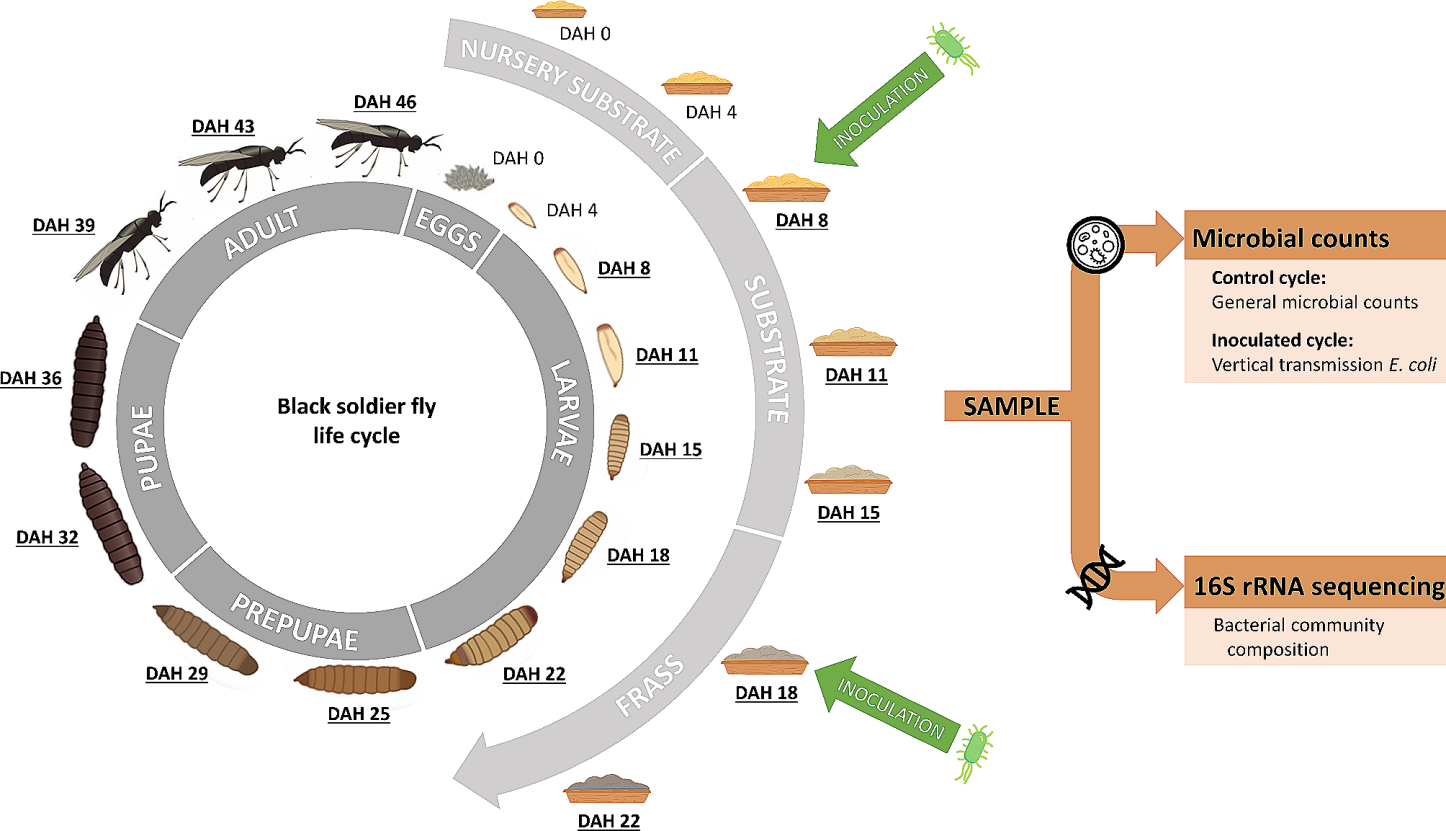
Life cycle of the black soldier fly. Sampling moments of the different life stages (larvae, prepupae, pupae and adults) and associated substrate and frass are indicated by underlining (DAH = the day after hatching). Green arrows indicate inoculation of the substrate/frass with *E. coli*

*Escherichia coli* strain BW25113 SX4 was obtained from the Centre of Microbial and Plant Genetics (Leuven, Belgium). To avoid overgrowth of background bacteria causing difficulties in the detection of *E. coli* on the used medium (observed in preliminary experiments, data not shown), a kanamycin-resistant *E. coli* BW25113 strain, obtained from the Centre of Microbial and Plant Genetics (KU Leuven, Leuven, Belgium, genetic modification described in [35]) and confirmed for its kanamycin resistance through preliminary tests (data not shown), was used. The bacterial strain was cultivated overnight in Luria-Bertani broth (10 g/L tryptone (VWR International, Leuven, Belgium), 5 g/L yeast extract (VWR International), 10 g/L NaCl), supplemented with kanamycin (50 µg/mL; Thermo Fisher Scientific, Merelbeke, Belgium) and incubated at 37 °C on a shaking plate (Ecotron, Infors HT, Velp, the Netherlands) at 150 rpm. The overnight culture was centrifuged (10 min, 3060 g; Multifuge 3 S, Heraeus, Houthalen-Helchteren, Belgium) and the pellet was resuspended in peptone physiological salt solution (0.1% peptone (Biokar Diagnostics, Beauvais, France), 0.85% NaCl) to a density of 3.0 McFarland units (measured with a DEN-1 McFarland 166 Densitometer, Grant instruments, Cambridge, UK) corresponding to approximately 9.0 log cfu/mL (determined in preliminary experiments, data not shown). Before inoculation of *E. coli*, the inoculum was plated on Rapid’*E. coli* 2 medium (Bio-Rad Laboratories, Temse, Belgium) supplemented with kanamycin (50 µg/mL), and incubation at 44 °C for 24 h to confirm its viability and inoculation level. For the inoculated cycle, 50 mL of *E. coli* suspension was added to the substrate on DAH 8 and 18 to obtain a concentration of approximately 7.0 log cfu/g of *E. coli*, as shown in Fig. 1.

### Sampling

Figure 1 shows the different sampling moments of the BSF life stages and associated substrate and frass. For both the control cycle and the cycle inoculated with *E. coli*, samples of the substrate were taken at DAH 8, 11 and 15. At DAH 18, which corresponds to the moment when BSF larvae are usually harvested in rearing facilities, and DAH 22, samples were considered as frass. Substrate and frass samples were collected after gently mixing with a tablespoon and randomly taking about 5 g of substrate or frass. After DAH 22, no samples could be taken from the frass, as prepupae began to crawl out at DAH 23–24. Samples of BSF were collected by sieving (separation from the substrate of frass in case of larvae) and randomly picking up by forceps about 5 g of insects at five sampling moments during larval stage (DAH 8, 11, 15, 18 and 22), two during prepupal stage (DAH 25 and 29), two during pupal stage (DAH 32 and 36) and three during adult stage (DAH 39, 43 and 46). From the control cycle or inoculated cycle, 2 or 3 technical replicates, respectively, were taken from the same container. To exclude microorganisms present on the exoskeleton of BSF, a disinfection treatment was used as described in previous studies [26, 36]. In short, the insects were first washed under running tap water, followed by one washing step with a 70% ethanol solution and two washing steps with sterile distilled water, each performed on a laboratory shaker (Unimax 1010, Heidolph, Swabach, Germany) at 200 rpm for 1 min.

### Analysis of microbial counts

As shown in Fig. 1, all samples were subjected to analyses of microbial counts, which were performed according to the ISO-standards for microbial analyses of food and feed, as compiled by [37], except for the media used for enumeration of *E. coli*. For each sample, 5 g were suspended in 45 g of peptone physiological salt solution to obtain a primary dilution. For BSF samples, the insects were aseptically grinded in the liquid during 1 min using a home-type kitchen mixer (Ergomixx, Bosch, Gerlingen, Germany). In between samples, the mixer was rinsed with water to remove the remains of the mixed sample, followed by a disinfection step with ethanol and flame sterilisation. Subsequently, primary dilutions were homogenised for 1 min in a stomacher (BagMixer, Interscience, Saint Nom, France). Tenfold dilution series were prepared from the primary dilution and plated on different media. Samples from the control cycle were plated on Plate Count Agar (PCA; Biokar Diagnostics) for determination of total (aerobic) viable counts after incubation at 30 °C for 72 h. The number of *Enterobacteriaceae* was determined on Violet Red Bile Glucose agar (VRBG; Biokar Diagnostics) followed by incubation at 37 °C for 24 h. Lactic acid bacteria were counted on de Man, Rogosa and Sharpe agar (MRS; Biokar Diagnostics) after incubation at 30 °C for 72 h. Fungi were enumerated after spreading on Dichloran Rose Bengal Chloramphenicol agar (DRBC; VWR International) and incubation at 25 °C for 5 days. Aerobic bacterial endospores were determined by first applying a heat shock (80 °C for 10 min) on the primary dilution, followed by preparation of a tenfold dilution series, plating on PCA and incubation at 37 °C for 48 h. The chromogenic Rapid’*E. coli* 2 medium (Bio-Rad Laboratories) supplemented with kanamycin (50 µg/mL), was used for enumeration of *E. coli* after incubation at 44 °C for 24 h. For samples from the inoculated cycle, the total viable counts and counts for the *Enterobacteriaceae* and *E. coli* were determined as described above. All plating was performed in duplicate and results of the microbial counts were calculated as the mean ± standard deviation, expressed in log cfu/g.

### DNA extraction and 16S rRNA gene amplicon sequencing

Samples from both the control and inoculated cycle were subjected to 16s rRNA sequencing. Starting material for DNA extraction was obtained by centrifuging (10 min, 3500 gMultifuge 3S, Heraeus) 5.0 mL of the primary dilution of each sample, discarding the liquid and remaining the pellet. Total genomic DNA was directly extracted from 50–250 mg of the pellet using the E.Z.N.A.® Soil DNA Kit (Omega Bio-Tek, Norcross, GA, USA), following the manufacturer’s instructions. A Nanodrop device (ND-1000, Isogen Life Science, Utrecht, the Netherlands) was used for determination of the DNA concentration and purity of the extract. Next, the V4 region of the 16S rRNA gene of extracted DNA was subjected to PCR amplification using barcoded primers 515F (5’-GTG CCA GCM GCC GCG GTA A-3’) and 806R (5’-GGA CTA CHV GGG TWT CTA AT-3’) [38] by Novogene Bioinformatics Technology (Cambridge, UK). PCR amplification contained 15 µL of Phusion® High-Fidelity PCR Master Mix (New England Biolabs, Ipswich, MA, United States), 0.2 µM of each primer and 10 ng template DNA. The PCR amplification program consisted of initial denaturation at 98 °C for 1 min, followed by 30 cycles of denaturation at 98 °C for 10 s, annealing at 50 °C for 30 s and extension at 72 °C for 30 s, and ended by final extension at 72 °C for 5 min. PCR products of proper size were selected through 2% agarose gel electrophoresis, purified using a Qiagen Gel Extraction Kit (Qiagen, Hilden, Germany), and equimolar concentrations for each sample were pooled into a sequencing library by Novogene Bioinformatics Technology. The library was checked with Qubit 2.0 Fluorometer (Thermo Fisher Scientific) and real-time PCR for quantification and with Bioanalyzer 2100 system (Agilent) for library size distribution detection, and was sequenced using the Illumina NovaSeq 6000 platform of Novogene Bioinformatics Technology.

Sequences were obtained from Novogene Bioinformatics Technology as demultiplexed FASTQ file, with barcodes and primer sequences removed. Paired-end reads for the V4 sequences were merged using USEARCH (v11.0.667) to form consensus sequences for individual samples [39], permitting a maximum of 10 mismatches within the overlap region. Subsequently, V4 region sequences were truncated at the 250th base to eliminate low-quality bases at extremities. Quality filtering was done using the expected numbers of errors based on the recalculated Phred scores of the merged reads [40], where reads under 250 bp or exceeding a total expected error threshold of 0.5 were excluded through the fastq_filter command in USEARCH. Reads under 250 bp or exceeding a total expected error threshold of 0.5 were excluded using USEARCH. Next, Mothur’s (v1.48.0) commands ‘classify.seqs’ and ‘remove.lineage’ were used in combination with the 16S ITGDB database, a database for taxonomic classification of 16S rRNA sequences integrating sequences from RDP (version NO.18 trainset), SILVA (version 138) and Greengenes (version 13_8) [41], to eliminate potential mitochondrial, chloroplast and other non-target sequences. The remaining bacterial sequences were clustered into zero-radius operational taxonomic units (zOTUs [42], also known as amplicon sequence variants (ASVs) [43]) using the UNOISE3 algorithm as implemented in USEARCH [40]. Only zOTUs with a minimum abundance of eight reads were retained, and chimeric sequences were removed for further analysis [39]. The taxonomic origin of bacterial zOTUs was determined using the SINTAX algorithm in USEARCH, based on the 16S ITGDB database.

### Data analysis and statistical analysis

Microbial counts of samples of the substrate, frass and BSF life stages within both the control and inoculated cycle were subjected to statistical analysis using *JMP Pro* 17.0.0 software from SAS. For data with normal distribution (assessed using Shapiro-Wilk test) and equal variances (assessed using Brown-Forsythe test), one-way analysis of variance (ANOVA) was applied, followed by a Tukey HSD post-hoc test. In case of non-normally distributed data, a non-parametric Kruskal-Wallis analysis, followed by a Wilcoxon each pair test was performed. Welch’s ANOVA with Steel-Dwass all pairs post-hoc test was used in case of unequal variances. For plate counts below the detection limit, the detection limit itself was used for statistical analysis.

After processing the sequencing data, zOTUs with a relative abundance below the 0.1% threshold per sample were discarded for further analysis. Next, samples of the same substrate type (inoculated or not) and sampling time (DAH) were pooled. Taxonomic assignments were generally considered reliable when bootstrap confidence values exceeded 0.80. Furthermore, the identity of the most important zOTUs was verified with a BLAST search of a representative sequence against type materials in GenBank [44]. After quality filtering and rare sequences removal, remaining bacterial zOTUs were used for subsequent analysis. Sequence data have been deposited in the NCBI Sequence Read Archive (SRA) under Bioproject PRJNA1068952.

Alpha diversity metrics, including observed richness (also called bacterial richness [45]), Shannon diversity index (exponential of the Shannon entropy) and Simpson’s diversity index, were calculated for each sample type using the *phyloseq* package in R (v4.3.0) [46, 47]. Statistical differences within each diversity index between the substrate and frass and between the different BSF life stages were determined using *JMP Pro* 17.0.0 software from SAS. An independent samples t-test was performed in case of normality and homoscedasticity assumptions, which were evaluated using a Shapiro-Wilk test and Levene’s test, respectively. If normal distribution was not confirmed, a nonparametric Mann-Whitney U test was performed, and Welch’s t-test was used in case of unequal variances. For comparison of each α-diversity metric between BSF life stages, a nonparametric Kruskal-Wallis analysis was performed in case of non-normal distribution, followed by a Wilcoxon each pair test. In case of unequal variances, a Welch’s ANOVA with a Steel-Dwass all pairs post-hoc test was applied.

Additionally, bacterial composition similarities between sample types were visualised in a Principal Coordinates Analysis (PCoA) ordination plot based on Bray-Curtis similarities of Hellinger-transformed relative abundance data, using the *vegan* and *phyloseq* package in R. To assess significant differences in bacterial community composition between different sample types, permutational multivariate analysis of variance (PERMANOVA) was performed using the *vegan* package in R. A significance level of α = 0.05 was considered for all statistical analyses.

## Results and discussion

### Dynamics of general microbial counts throughout a BSF rearing cycle (control cycle)

**Fig. 2:**
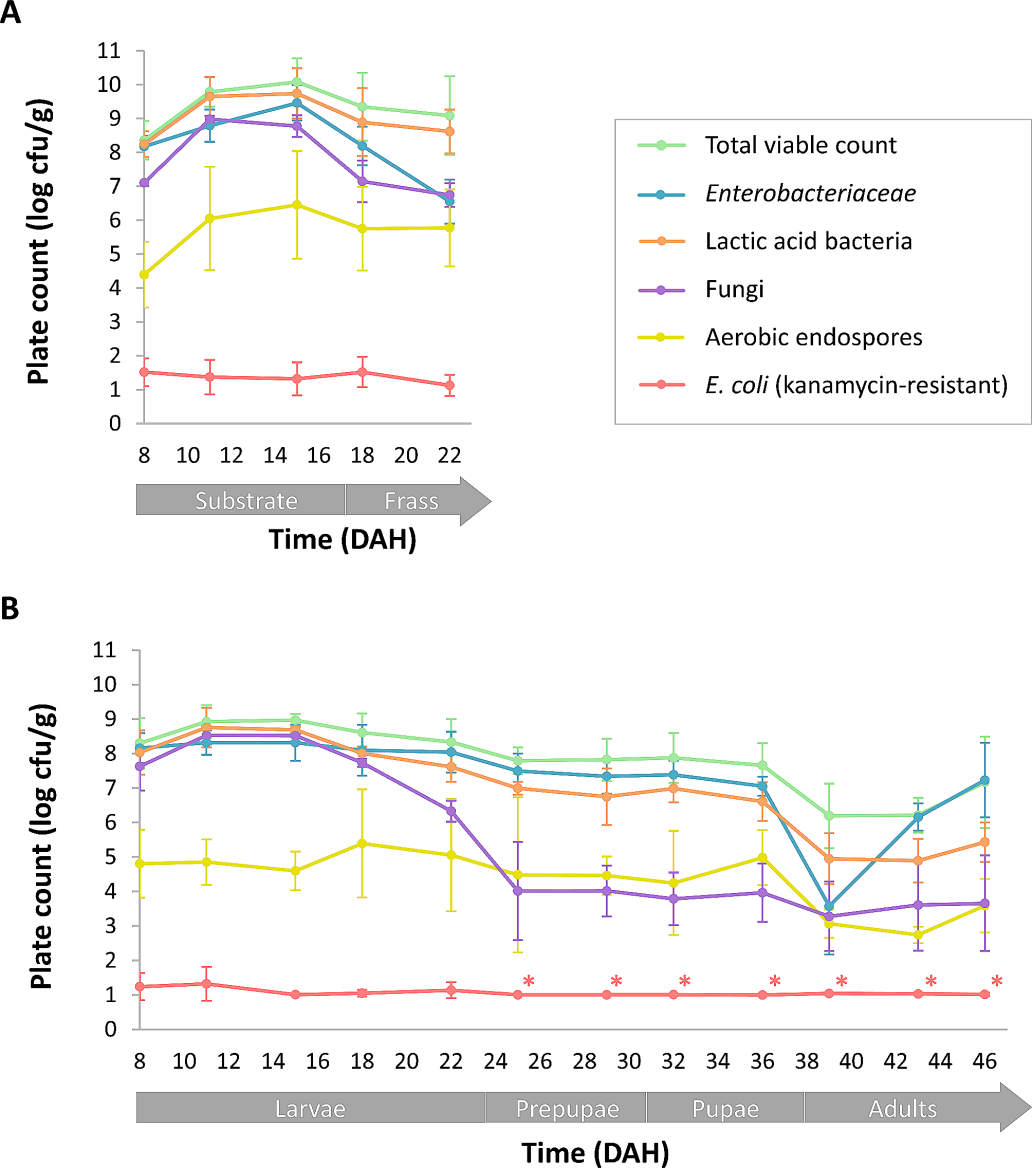
Microbial counts of the control cycle throughout the time (DAH = day after hatching) **(A)** substrate (chicken feed) and frass and **(B)** different BSF life stages. Results are presented as the mean of 6 replicates (*n* = 3 cycle repetitions × 2 technical repetitions) ± standard deviation. The symbol * represents microbial counts below the detection limit (1.0 log cfu/g)

Microbial plate counts were monitored in the substrate, frass and different BSF life stages of the control cycle, starting from the introduction of 8-day-old larvae to the freshly prepared chicken feed (DAH 8), and continued until the end of the adult stage at DAH 46. Average microbial counts are shown in Fig. 2. Supplementary Table S1 provides more detailed values.

#### Microbial counts of the substrate and frass

General microbial counts for the substrate and frass are shown in Fig. 2A. Upon introduction of the 8-day-old larvae and a fraction of the nursery substrate, high initial counts above 8.0 log cfu/g were observed for the total viable count, *Enterobacteriaceae* and lactic acid bacteria within the substrate. Fungal counts resulted in 7.1 ± 0.1 log cfu/g. These high counts are attributed to the substantial microbial load inherent to the nursery substrate or the exoskeleton of the larvae when transferred from the nursery substrate to the freshly prepared chicken feed substrate, or to larval excrements containing the gut microbiota of the larvae. Over the initial three rearing days (DAH 8–11), all general plate counts significantly increased, most of them with more than one log-unit. Indeed, the high water activity (a_w_), determined as 0.96 to 0.97 in another study using a comparable mix of chicken feed and water [26], and the availability of nutrients in the substrate (not determined in this study) at the beginning of the rearing cycle create optimal microbial growth conditions [48]. For the total viable count, *Enterobacteriaceae* and lactic acid bacteria, the highest counts within the substrate were observed at DAH 15, with counts over 9.5 log cfu/g for each parameter. When considering the frass from DAH 18, the counts of the *Enterobacteriaceae* were reduced, ending at 6.6 ± 0.6 log cfu/g at DAH 22, while high total viable counts (9.1 ± 1.2 log cfu/g) and counts for the lactic acid bacteria (8.6 ± 0.7 log cfu/g) were still observed for the frass at DAH 22. These microbial counts were comparable to other studies reporting microbial counts for BSF frass, although different substrates were administered to the larvae [26, 28, 30]. Possible explanations for the slight decrease in microbial counts can be found in changes in the pH or a_w_ of the substrate and frass (not determined in this study), which were previously considered as important factors determining microbial growth in BSF substrates, nutrient depletion, increased production of antimicrobial compounds by the growing larvae or a combination of these factors [5]. Fungal counts exhibited its highest values in the substrate at DAH 11 (8.2 ± 0.3 log cfu/g), followed by a gradual reduction to 6.7 ± 0.4 log cfu/g in the frass at DAH 22. These fungal counts were higher compared to the counts reported for frass from three large scale facilities (3.6–4.8 log cfu/g), but lower compared to fungal counts reported for frass of BSF larvae reared on food waste (7.5–8.7 log cfu/g) [26, 28]. Aerobic endospore counts showed a comparable trend to the total viable count and counts for lactic acid bacteria, but consistently remained 3 to 4 log-units lower than the total viable counts. These endospore counts were comparable to those reported for BSF frass in another study (4.2–7.0 log cfu/g) evaluating the microbial counts during BSF larvae rearing on organic waste streams on both laboratory and large scale [26].

To the best of the authors’ knowledge, this study is one of the first integrating comprehensive temporal dynamics of various general microbial counts of the substrate and frass during BSF larvae rearing. Previous studies predominantly focused on microbial counts within the initial substrate and/or residual frass [5, 26, 29, 30, 49, 50], or limited their study to selected bacterial parameters at multiple time points throughout the rearing process [28].

Beyond general microbial counts, *E. coli* counts were determined within the substrate and frass of the control cycle to define a baseline for the following inoculated cycle. To ensure proper enumeration of distinctive *E. coli* colonies, kanamycin (50 µg/mL) was supplemented to the chromogenic Rapid’*E. coli* 2 medium, preventing outgrowth of background microorganisms and innate *E. coli* of BSF. Consequently, it should be noted that only kanamycin-resistant *E. coli* was counted, consistently resulting in average counts below 1.5 log cfu/g for both the substrate and frass, as shown in Fig. 2A.

#### Microbial counts of BSF life stages

Figure 2B shows an overview of the general microbial counts observed throughout the BSF life stages of the control cycle. From the beginning of the experiment, 8-day-old larvae exhibited a high microbial load, characterised by a total viable count and counts for the *Enterobacteriaceae*, lactic acid bacteria and fungi between 7.6 and 8.3 log cfu/g and aerobic endospore counts of < 4.8 ± 1.0 log cfu/g. These counts were common when comparing to other studies reporting microbial counts for 8-day-old larvae [5, 7, 26]. In contrast to the observed increase in general counts within the substrate by DAH 15, all microbial counts for the larvae remained relatively constant until DAH 22, except for the fungi. Table 1 shows the average microbial counts for the different BSF life stages. During the larval stage, the total viable counts and counts for the *Enterobacteriaceae* and lactic acid bacteria varied within a maximum of 1.2 log-units, suggesting that the larval age had limited influence on its microbial load. An exception of stable microbial counts was observed for the fungal counts, which were reduced to 6.3 ± 0.3 log cfu/g in the older larvae (DAH 22), demonstrating the predominance of bacteria over fungi in the older larvae. This reduction in fungal counts related to larval age was also reported in a previous study, with 5.4–7.1 log cfu/g for 7-day-old larvae and 3.1–3.8 log cfu/g for 21-day-old larvae [26]. Although average counts for the bacterial endospores consistently varied between 4.6 and 5.4 log cfu/g within the larvae, relatively large standard deviations were observed.

**Table 1:** Average microbial counts of the different BSF life stages in the control cycle. Results are presented as the mean of 30 (*n* = 5 sampling moments x 3 cycle repetitions x 2 technical repetitions), 12 (*n* = 2 × 3 × 2), 12 (*n* = 2 × 3 × 2) and 18 (*n* = 3 × 3 × 2) replicates for the larval, prepupal, pupal and adult stage, respectively, ± standard deviation

	Average microbial counts (log cfu/g)																	
	Total viable count			*Enterobacteriaceae*			Lactic acid bacteria			Aerobic endospores			Fungi			*E. coli* (kanamycin-resistant)		
Larvae	8.6	±	0.6^c^	8.2	±	0.5^c^	8.2	±	0.6^c^	< 5.0	±	1.1^b^	7.7	±	0.9^b^	< 1.2	±	0.3^a^
Prepupae	7.8	±	0.5^b^	7.4	±	0.5^b^	6.9	±	0.6^b^	< 4.5	±	1.6^b^	< 4.0	±	1.1^a^	< 1.0	±	0.0^a^
Pupae	7.8	±	0.7^b^	7.2	±	0.4^b^	6.8	±	0.5^b^	4.6	±	1.2^b^	3.9	±	0.8^a^	< 1.0	±	0.0^a^
Adult	6.5	±	1.0^a^	< 5.4	±	2.0^a^	> 5.1	±	0.7^a^	< 3.1	±	0.6^a^	< 3.5	±	1.2^a^	< 1.0	±	0.0^a^

^a, b,c^ Means of samples of different life stages of BSF with the same letter in superscript in the same column do not differ significantly (*p* ≥ 0.05)

Although the larval BSF stage often receives most interest due to its importance in industrial applications [26, 28, 30], insight in the microbial counts dynamics of subsequent life stages is essential to completely understand the microbial community throughout BSF rearing. Notably, the transition from larval to prepupal stage correlated with a reduction of all general plate counts (Fig. 2B). Indeed, comparison of average counts for the larval and prepupal stage (Table 1) indicated significantly lower counts for the total viable count (*p* = 0.002), counts for the *Enterobacteriaceae* (*p* = 0.003), lactic acid bacteria (*p* < 0.001) and fungi (*p* < 0.001) for the prepupae compared to the larvae. The prepupae crawling out of the substrate, associated with cessation of feeding and starting to empty their digestive tract towards pupation [51], is supposed to be linked to the reduction of the microbial counts. Remarkably, aerobic endospore counts remained consistent across the larval, prepupal and pupal stage. This suggests that sporulation and endospore germination were minimally affected by the transition from larvae to pupae, or that endospores remained present and were not killed across these life stages. Within both the prepupal and pupal stage, stability persisted for all microbial counts, with minimal variations (not exceeding 0.4 log-units) observed for the total viable count, *Enterobacteriaceae*, lactic acid bacteria and fungi.

As expected, the transition from pupae (DAH 36) to adults (DAH 39) marked the most distinct change in microbial counts, characterised by a reduction of all microbial parameters, as illustrated in Fig. 2B. A reduction of 1.5–2 log cfu/g was observed for the total viable count, lactic acid bacteria and aerobic endospores, while the average count for the *Enterobacteriaceae* was substantially reduced with 3.5 log-units to > 3.6 ± 1.4 log cfu/g. Holometabolous insects, like the BSF, undergo complete metamorphosis, entailing a complete gut microbial community remodelling. Pupation involves purging of the gut content, followed by reshaping the gut microbiota according to the digestive needs of the fly [1, 33, 34]. This could declare the dynamic fluctuations of the microbial counts during transformation to flies. It is conceivable that microorganisms recolonised the emerged flies after contact with the remaining puparia, the water source or other previously emerged flies. Notably, further development of the flies again led to elevated counts for the *Enterobacteriaceae * (7.2 ± 1.1 log cfu/g), which emerged as the most dominant group within the total viable count (7.2 ± 1.3 log cfu/g) during the adult stage. As given in Table 1, only fungal counts did not significantly differ between the pupal and adult stages (*p* = 0.688). Finally, consistent with the substrate, kanamycin-resistant *E. coli* counts remained below 1.3 log cfu/g for all sampling moments across all life stages.

In summary, these findings illustrated the dynamic nature of the microbial counts throughout the BSF rearing cycle, highlighting the substantial impact of metamorphosis on the microbial load.

### Vertical transmission of *E. coli* throughout a BSF rearing cycle (inoculated cycle)

**Fig. 3:**
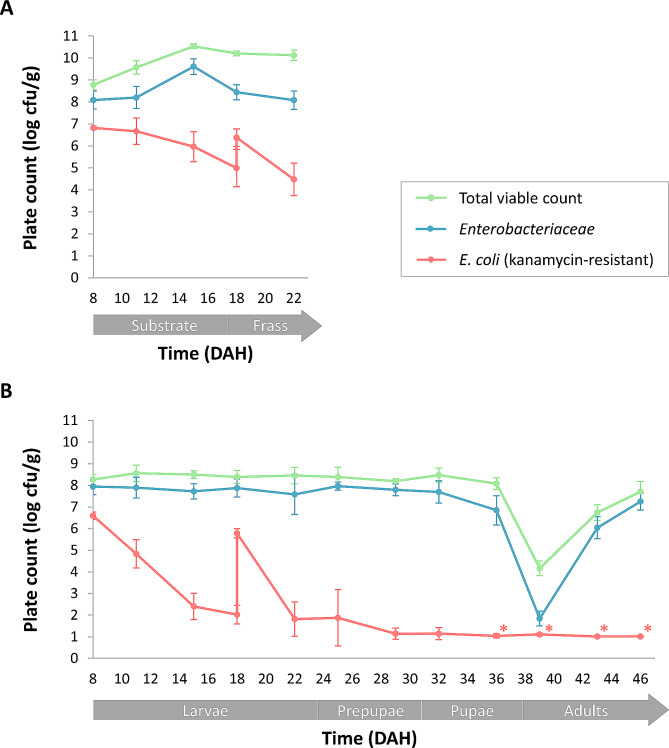
Microbial counts of the inoculated cycle throughout the time (DAH = day after hatching) **(A)** substrate (chicken feed inoculated with kanamycin-resistant *E. coli* at DAH 8 and 18) and frass and **(B)** different BSF life stages. Results are presented as the mean of 9 replicates (*n* = 3 cycle repetitions × 3 technical repetitions) ± standard deviation. The symbol ‘*’ represents microbial counts below the detection limit (1.0 log cfu/g)

To assess the vertical transmission of *E. coli*, kanamycin-resistant *E. coli* counts were monitored in the substrate (inoculated with kanamycin-resistant *E. coli* at DAH 8 and 18), frass and different BSF life stages of the inoculated cycle. As with the control cycle, analyses started after introduction of 8-day-old larvae to the inoculated substrate (DAH 8) and lasted until the adult stage at DAH 46. Throughout this period, the total viable counts and counts for the *Enterobacteriaceae* were followed up as well, and results are shown in Fig. 3.

#### *E. coli* in the substrate and frass

At the onset of the rearing cycle, the substrate was inoculated with *E. coli* at a concentration of 6.8 ± 0.1 log cfu/g, as illustrated in Fig. 3A. This approached the targeted inoculation level of 7.0 log cfu/g. As the counts for *E. coli* in the control substrate (Fig. 2A) were consistently below 1.5 log cfu/g, about all *E. coli* detected in the substrate originated only from the initial inoculation. Similar to the control cycle, introduction of 8-day-old larvae, along with a small amount of nursery substrate, resulted in total viable counts and counts for the *Enterobacteriaceae* above 8.0 log cfu/g within the substrate. These high counts were attributed not only to the inoculation of *E. coli* in the substrate, but mainly to the substantial microbial load of the nursery substrate and on the larvae. Again, the highest counts in the substrate were observed at DAH 15 for both the total viable count (10.5 ± 0.1 log cfu/g) and the *Enterobacteriaceae* (> 9.6 ± 0.4 log cfu/g). In contrast, *E. coli* counts were significantly reduced in the frass to 5.0 ± 0.8 log cfu/g by DAH 18 (*p* < 0.001). A second inoculation at DAH 18 was performed to elevate the *E. coli* counts in the larvae at the end of the larval stage, elevating *E. coli* levels in the frass to 6.4 ± 0.4 log cfu/g. Again, this second inoculation did not impact the total viable count and counts for the *Enterobacteriaceae*, as these counts were at least one log-unit above the inoculation level of 7.0 log cfu/g. While the total viable counts only slightly decreased to 10.1 ± 0.2 log cfu/g by DAH 22, average counts for the *Enterobacteriaceae* were reduced to 8.1 ± 0.4 log cfu/g in the frass, which was remarkably higher compared to the control cycle. As *E. coli* counts were at least 3.0 log-units lower than the *Enterobacteriaceae* counts, the reason behind these elevated counts in the inoculated cycle remains unclear. Intriguingly, a statistically significant reduction of *E. coli* counts was faster after the second inoculation compared to the initial inoculation, with counts reduced to 4.5 ± 0.7 log cfu/g in the frass at DAH 22 (*p* < 0.001). The fact that older larvae might have a stronger, more stable microbiome which could provide better resistance against foreign microorganisms and compete with *E. coli*, might be a possible hypothesis for this observation, but scientific evidence is still needed for substantiation.

Notably, if this frass would be intended to be used as plant fertiliser or soil amendment, thermal treatment is inevitable to mitigate the *E. coli* counts. A heat treatment at 70 °C for 60 min, as imposed by EU Regulation (EU) No. 2021/1925 for insect frass, should be appropriate to reduce *Enterobacteriaceae*, and consequently also *E. coli*, to below 1.0 log cfu/g, as previously reported [50].

#### Vertical transmission of *E. coli* across BSF life stages

Figure 3B illustrates the microbial counts observed across the different BSF life stages of the cycle inoculated with *E. coli*. Preceding their introduction into the inoculated substrate, 8-day-old larvae exhibited a comparable microbial load as for the control cycle, with an average total viable count of 8.2 ± 0.4 log cfu/g and *Enterobacteriaceae* counts of 7.8 ± 0.5 log cfu/g. Upon introduction in the inoculated substrate, minimal changes in these microbial counts were noted, with an average total viable count and counts for the *Enterobacteriaceae* of approximately 8.0 log cfu/g.

Intriguingly, introduction of the 8-day-old larvae into the inoculated substrate resulted in contaminated larvae with an average *E. coli* count of 6.6 ± 0.2 log cfu/g (Fig. 3B) within one hour (i.e. approximate time between inoculation of the substrate and sampling), closely approaching the level found within the inoculated substate. Since young larvae from the control cycle showed *E. coli* counts below 1.2 log cfu/g (Fig. 2B), *E. coli* counts in the larvae entirely originated from ingestion of the inoculated substrate. Although the *E. coli* counts in the frass remained relatively high at DAH 18, the larvae were able to reduce the *E. coli* counts within their bodies to 2.4 ± 0.6 log cfu/g at the moment they are usually harvested for further processing into animal feed. Again, the second inoculation of *E. coli* at DAH 18, aiming to evaluate vertical transmission of *E. coli* from the end of the larval stage to the (pre)pupal and adult stages did not affect the total viable count and counts for the *Enterobacteriaceae* of the larvae. However, *E. coli* counts increased to 5.8 ± 0.2 log cfu/g within the larvae. Subsequently, the larvae rapidly reduced these high *E. coli* counts to < 1.8 ± 0.8 log cfu/g by DAH 22 (*p* < 0.001). The fast reduction of *E. coli* in the larvae can be attributed to their efficient immune system, involving several cellular and humoral mechanisms to counter bacterial infections, which is crucial for BSF larvae to survive on substrates with high bacterial load [52, 53] Indeed, antimicrobial activity of BSF larvae against *E. coli* has been previously reported [52–55]. Mechanisms such as phagocytosis, lysozyme activity, antimicrobial peptide production and the phenoloxidase system likely contribute to counteracting bacterial threats [52], but also its gut microbiota can contribute to the antimicrobial activity of the larvae [56, 57]. Besides the larval immune system, pathogen reduction may be ascribed to passage through the acidic and alkaline zones of the larval gut, especially the midgut characterised by a pH below 3 [17, 21]. Nevertheless, resistance of *E. coli* against extreme acid stress, resulting in survival at pH 2 to 3 for hours, had been reported [58, 59].

As illustrated in Fig. 3B, the total viable counts remained relatively stable throughout the larval, prepupal and the pupal stage, with variations of no more than one log-unit, ranging from 8.1 to 8.6 log cfu/g. Similarly, *Enterobacteriaceae* counts exhibited minimal variation until the pupal stage, ranging from 6.9 to 8.0 log cfu/g. Further reduction of *E. coli* to < 1.1 ± 0.3 log cfu/g continued during the prepupal stage. In contrast to the larvae, studies on the immune system of BSF (pre)pupae or adults are scarce, but it is plausible that similar mechanisms occur in the (pre)pupal stage.

Similar to the control cycle, metamorphosis to adult stage was marked by a significant reduction of microbial counts. During this transition (DAH 36 to DAH 39), the total viable counts were significantly reduced to 4.2 ± 0.3 log cfu/g (*p* = 0.002). Notably, counts for the *Enterobacteriaceae* showed an even more substantial reduction of approximately 5 log-units to < 1.8 ± 0.3 log cfu/g (*p* = 0.002). During the adult stage, a new increase of microbial counts was observed, due to the remodelling of the gut microbiota. In addition, the flies will be recolonised by microorganisms immediately after emergence, as they start flying and having contact to surfaces, water and the remaining puparia. The total viable counts and *Enterobacteriaceae* counts exceeded 7.0 log cfu/g at the end of the adult stage (DAH 46). Remarkably, from the end of the pupal stage (DAH 36) and during the entire adult stage, *E. coli* counts consistently remained below the detection limit of 1.0 log cfu/g for all samples.

In conclusion, vertical transmission throughout one BSF life cycle was not observed here for *E. coli*. Consequently, using substrates contaminated with *E. coli* for rearing BSF larvae does not necessarily pose a microbiological safety problem for persistent contamination of the BSF colony. Given that vertical transmission of foodborne pathogens might be affected by several factors, such as the insect species, pathogen species and pathogen level, these findings can not be generalised. Hence, this study paved the way for further case studies on vertical transmission across consecutive life stages of BSF or other insect species. Furthermore, vertical transmission across more life cycles might be an interesting additional step towards gaining more insight in pathogen transmission processes and microbiological food or feed safety associated with BSF or other insects.

### Bacterial community composition throughout a BSF rearing cycle

Besides insight in the dynamics of culture-dependent microbial counts across consecutive BSF life stages and the associated substrate and frass, the bacterial community composition was explored by 16S rRNA gene sequencing of samples from both the control cycle and inoculated cycle. In total, 592 bacterial zOTUs were retained for subsequent analysis. Although vertical transmission of *E. coli* was not observed up to the adult stage, the potential impact of its introduction in the rearing cycle on the bacterial community of the BSF was investigated in this study.

#### Bacterial community composition of the substrate and frass

**Fig. 4:**
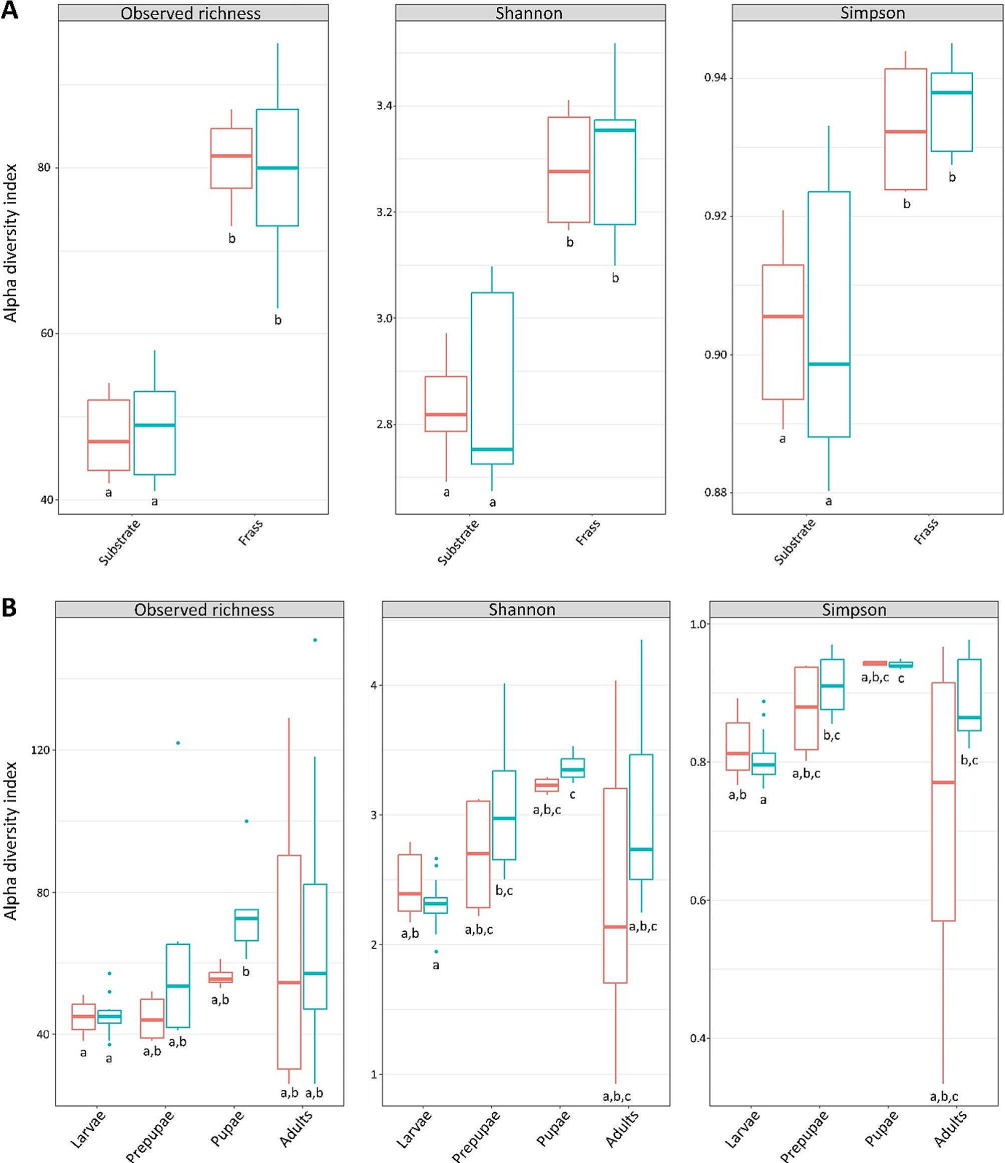
Bacterial diversity metrics (observed richness, Shannon diversity index and Simpson’s diversity index) of **(A)** substrate and frass and **(B)** different BSF life stages. Boxplots in red and blue represent the control and inoculated cycle, respectively. Means of samples of different sample type or inoculation condition with the same letter below the boxplots do not differ significantly (*p* ≥ 0.05)

To explore the bacterial diversity (α-diversity) of the substrate and frass, metrics such as the observed richness, Shannon diversity index and Simpson’s diversity index were calculated and visualised in Fig. 4A. Actual values for the diversity indices are given in Supplementary Tables S2 and S3. Both the Shannon and Simpson’s diversity index are often used to characterise species diversity in a community and take into account both richness and evenness of the identified species [45, 60]. The α-diversity of the frass was significantly higher than for the substrate of both the control and inoculated cycle, indicating that the larval rearing not only modulated the bacterial community of their environment [15, 61], but also increased its bacterial richness and diversity. In addition, inoculation of *E. coli* in the substrate did not influence the bacterial richness or diversity of the substrate and frass.

**Fig. 5:**
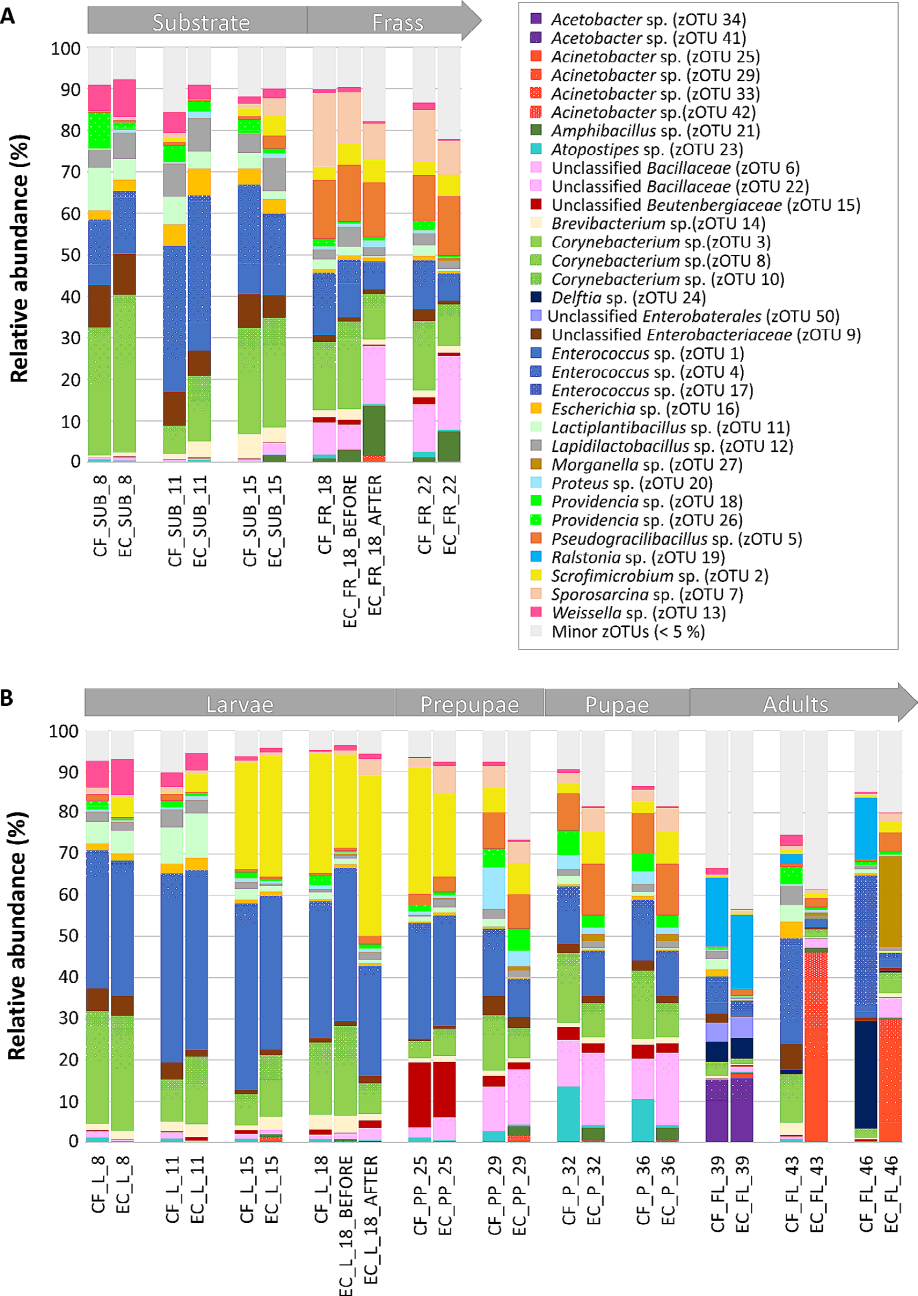
Relative abundances (%) of bacterial community composition present in **(A)** substrate (SUB) and frass (FR) and **(B)** different life cycles of BSF (larvae (L), prepupae (PP), pupae (P) and adults (FL)), reared on chicken feed (CF, control cycle) and chicken feed inoculated with kanamycin-resistant *E. coli* (EC, inoculated cycle). Numbers in the sample name represent the sampling time (DAH). Larval samples from DAH 22 were not analysed. Results are calculated as mean values of two (*n* = 2) or three (*n* = 3) extracts for the control cycle and inoculated cycle, respectively. zOTUs with a mean relative abundance of less than 5% for all samples were grouped in the “Minor zOTUs (< 5%)” category

The relative abundances of the zOTUs found in the substrate and frass samples from both the control and inoculated cycle are shown in Fig. 5A. Genera identified within both substrate and frass samples belonged to three phyla: *Actinomycetota*, *Bacillota* and *Pseudomonadota*. In the substrate, the genera *Enterococcus* (zOTU 1, 4 and 17) and *Corynebacterium* (zOTU 3, 8 and 10) were predominant, constituting over 40% of the total abundance in all substrate samples for both cycles. These genera were previously reported as commonly present in all kinds of substrates associated with BSF larvae, including chicken feed [16, 62, 63]. The prevalence of the *Lactobacillales* order in all substrate samples (> 32%) could explain the high counts of the lactic acid bacteria in Fig. 2A. Similarly, high relative abundances of zOTUs belonging to the lactic acid bacteria, such as *Lactiplantibacillus* sp. (zOTU 11; 2–10%) and *Weissella* sp. (zOTU 13; 2–9%), were also reported for chicken feed in other studies [62, 63]. In addition, members of *Lactiplantibacillus* sp. were previously suggested to have the ability to defend the insect against fungal infections [64]. Despite the introduction of *E. coli* into the substrate of the inoculated cycle, the abundance of *Escherichia* (zOTU 16; 3–6%) did not significantly increase compared to the control cycle (2–5%), because both inoculated (kanamycin-resistant) *E. coli* and innate *E. coli* excreted by the larvae or already present in the substrate, were considered.

Furthermore, a notable shift between the bacterial community composition of the substrate and the frass was observed. *Bacillales* became the predominant order, constituting over 33% abundance in all frass samples. At genus level, *Enterococcus* sp. (zOTU 1, 4 and 17) and *Corynebacterium* sp. (zOTU 3, 8 and 10) exhibited declined prominence, while others, such as *Pseudogracillibacillus* sp. (zOTU 5; 11–14%) and *Sporosarcina* sp. (zOTU 7; 8–18%), emerged. These results align with previous studies that frequently reported *Bacillus*, *Corynebacterium*, *Lactobacillus*, *Morganella* and *Sporosarcina* as abundant genera in BSF frass [26, 28, 29]. The high abundance of *Pseudogracillibacillus* sp. is rather uncommon, but was previously reported in the frass of BSF larvae grown on chicken feed [61]. Moreover, certain members of the genera identified in the frass bacterial community, such as members of the genera *Bacillus*, *Enterococcus* and *Lactobacillus*, have been previously suggested to be recognised as plant growth-promoting rhizobacteria, contributing to improved crop productivity, stimulated plant growth and pathogen suppression [65–67]. The potential of BSF frass as organic plant fertiliser should be further investigated to understand the impact of the microbial community of frass on plant growth. Comparable to the substrate, minimal differences were observed between frass from the control and inoculated cycle. Additionally, an *Escherichia* sp. (zOTU 16) was found in low abundance (approximately 1%) in frass from both cycles, even after the second inoculation of *E. coli* at DAH 18. These low abundances can be explained by comparing the *E. coli* inoculation level (6.4 log cfu/g) to the high total viable count (10.2 log cfu/g) within the frass.

**Fig. 6:**
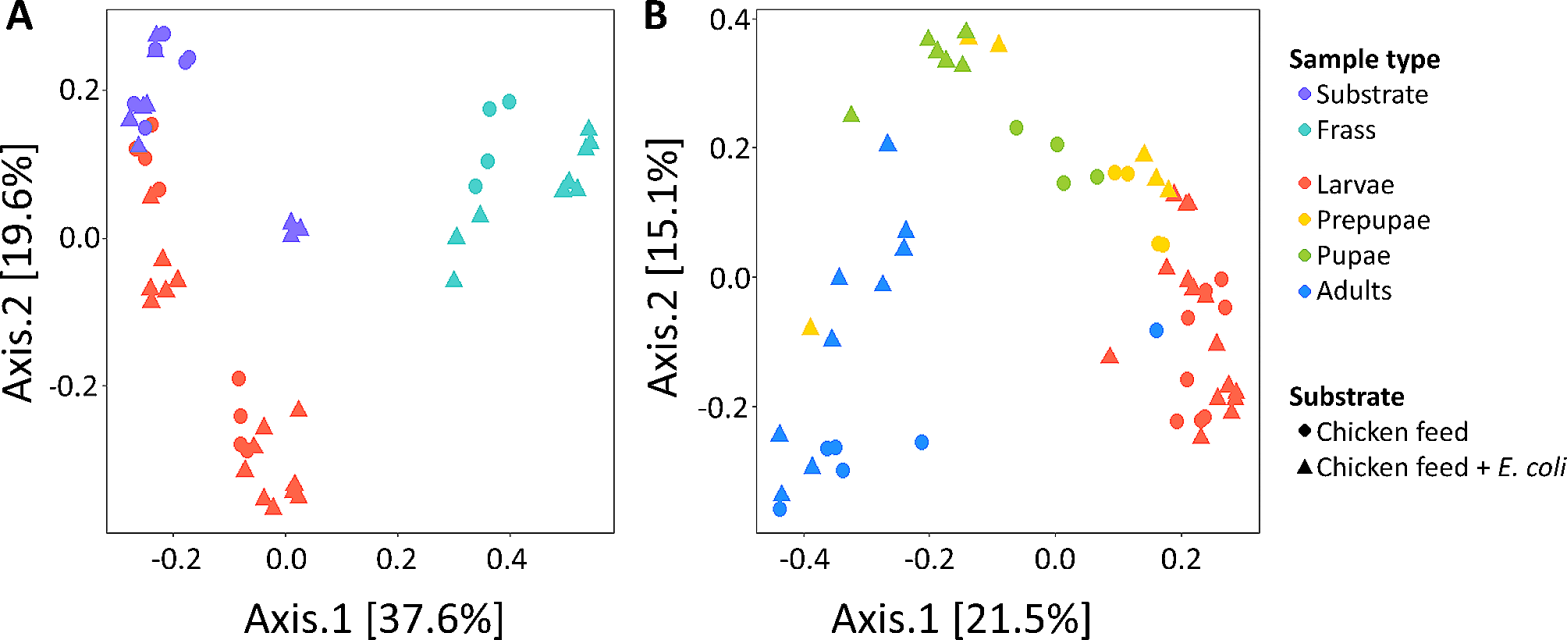
Principal Coordinates Analysis (PCoA) ordination plot composed of bacterial community data of **(A)** substrate, frass and larvae samples and **(B)** larvae, prepupae, pupae and adult samples from the control cycle (chicken feed, represented by circles) and the inoculated cycle (chicken feed + *E. coli*, represented by triangles). Samples of the same sample type are represented by the same colour. The distance between points illustrates their similarity: the smaller the distance between points, the more similar their bacterial community

The shift in bacterial composition between substrate and frass samples is also visualised through PCoA in Fig. 6A. These results revealed that the bacterial composition of the substrate and frass is mainly shaped by the sample type rather than introduction of *E. coli* into the substrate. This observation was confirmed by PERMANOVA, attributing 22% of the variability in bacterial community composition to the sample type (R^2^ = 0.22, *p* < 0.001), whereas inoculation of the substrate did not significantly change the bacterial composition (R^2^ = 0.03, *p* = 0.078). Hence, this suggests that BSF larvae are able to quickly get rid of the *E. coli* in the substrate, as observed for the *E. coli* counts before. In other studies, the impact of the larvae in shaping the bacterial community of the frass was suggested as a reason for the shift in bacterial community towards zOTUs related to the larvae [16, 68].

#### Bacterial community composition of BSF life stages

To compare the bacterial composition of the BSF life stages, bacterial diversity is visualised in Fig. 4B, while Fig. 5B shows the relative abundance of zOTUs within the life stages from both the control and inoculated cycle. Throughout the studied life cycle, three bacterial phyla, being *Actinomycetota*, *Bacillota* and *Pseudomonadota*, were relevant. These phyla, together with *Bacteriodota*, were previously reported as most dominant in BSF samples [18, 56]. During the larval stage, *Lactobacillales* emerged as the predominant order, explaining the high lactic acid bacteria counts in Fig. 2B. Most abundant representative of the lactic acid bacteria was *Enterococcus* sp. (zOTU 1, 4 and 17, > 26%), a common genus of commensal gut bacteria of insects, which contains species that are suggested to harbour carbohydrate-degrading enzymes and a nitrogen, hydrogen and sulphur metabolism [56, 68, 69]. Moreover, a positive correlation between BSF larvae reared on fermented substrates and the abundance of *Enterococcus* sp. was previously reported, with species of this genus harbouring genes involved in cellulose degradation, including endoglucanases and β-glucosidases [19]. Consequently, they might contribute to degradation of plant material in the substrate. In addition, some members of *Enterococcus* sp., such as *E. mundtii*, were previously linked to antimicrobial peptides production [69, 70], and might potentially play a role in the antimicrobial activity against *E. coli* in the inoculated cycle. Nevertheless, some members of the *Enterococcus* sp. are identified as pathogenic to human, such as *E. faecalis* and *E. faecium* [69, 71]. Similar to the substrate, *Corynebacterium* (zOTU 3, 8 and 10) represented another abundant bacterial genus, particularly in early larvae (DAH 8), constituting over 25% of the total abundance. Additional genera with over 5% abundance in the larvae at DAH 8 included *Lactiplantibacillus* (zOTU 11; 5–6%) and *Weisella* (zOTU 13; 7–9%), which were identified as most dominant in young larvae reared on chicken feed in other studies [16, 62].

Generally, the relatively low bacterial diversity of the larval samples (Fig. 4B) and the similarities between the bacterial composition of the (young) larvae and the substrate (Fig. 5A) can be explained by the fact that neonate larvae were reared on a comparable feeding substrate before DAH 8 and already adapted to the microbial community of the environment. It should also be noted that substrate samples at DAH 8 already contained a fraction of the nursery substrate due to introduction of the larvae. This suggests that horizontal transmission of the substrate bacterial community is essential to modulate the larval bacterial community to ensure quick adaptation to the rearing environment. While the bacterial composition of the substrate and the larvae strongly correlated, a shift of the larval bacterial community composition towards a profile distinct from that of the frass was observed over time, as illustrated in Fig. 5. This shift of the bacterial community during larval development, together with diversification, was also recently reported by other authors [16]. By DAH 15 and 18, the larval samples were predominantly composed of *Enterococcus* sp. (zOTU 1, 4 and 17), *Corynebacterium* sp. (zOTU 3, 8 and 10) and *Scrofimicrobium* sp. (zOTU 2), collectively defining over 70% of the total abundance. This low bacterial diversity is also reflected in Fig. 4B, representing the lowest bacterial diversity during the larval stage.

Although studies on the microbiota of BSF larvae substantially increased over the last years [17–20, 22, 25, 27, 62, 72], comparison between studies remains challenging, as various factors, such as the feeding substrate, larval age and gut region, affect the composition of the larval microbiota [15, 18]. Nevertheless, the genera *Corynebacterium*, *Enterococcus* and *Scrofimicrobium*, which were abundantly found in the larvae in this study, are ubiquitous in other studies and were even appointed as key members of the ‘core microbiota’ of BSF larvae, even though discussion on a clear definition of this term is still ongoing [18, 33, 62]. Other genera often included in the core microbiota are *Morganella* and *Providencia* [18]. In our study, *Providencia* sp. was found in all BSF samples, but always at an abundance below 6%, while, surprisingly, *Morganella* sp. was only found in BSF samples of the inoculated cycle. Both genera include specific species, such as *P. rettgeri* and *M. morganii*, which produce extracellular enzymes with a bacteriolytic effect against *E. coli* by degrading bacterial cell wall compounds [17].

However, in addition to the putative beneficial functions associated with members of the bacterial community of BSF larvae, several bacterial genera associated with BSF larvae include pathogenic species for humans or animals. For example, the families *Bacillaceae* and *Clostridiaceae* contain several pathogenic endospore-forming species, such as *B. cereus* and *Clostridium* spp., and were observed in low abundances for several larval samples in our study. These findings may lead to food safety issues when using BSF larvae as food or feed, but a culture-dependent approach is needed to reliably identify gut microorganisms on species level and examine their pathogenic nature [56].

In general, prepupal samples exhibited a more diverse set of bacterial genera, reflected by a higher Shannon and Simpson’s diversity index compared to the larval stage (Fig. 4B). The bacterial composition of prepupae samples of DAH 25 closely corresponded to this of the larvae, as illustrated in Fig. 6B. Nevertheless, PERMANOVA assigned 24% of the variability in bacterial community composition across all life stages to the sample type (R^2^ = 0.24, *p* < 0.001) and, to a lesser extent (9%), to inoculation of the substrate (R^2^ = 0.09, *p* < 0.001). *Enterococcus* (zOTU 1, 4 and 17, 27–28%) and *Scrofimicrobium* (zOTU 2, 20–31%) remained the most predominant bacterial genera of the prepupae at DAH 25. By DAH 29, bacterial diversification increased, with additional emerging genera, including *Proteus* (zOTU 20), *Providencia* (zOTU 18 and 26), *Pseudogracillibacillus* (zOTU 5), *Sporosarcina* (zOTU 7) and unclassified *Bacillaceae* (zOTU 6 and 22).

Further development to pupae retained stability in the bacterial composition, as illustrated by clustering of prepupal and pupal samples in Fig. 6B. Although the bacterial composition differed minimally, the α-diversity of the pupae continued to increase (Fig. 4B), which was also reported in another study [61]. This high bacterial diversity supports the decoupling hypothesis for holometabolous insect species, which assumes decoupling of the bacterial community of the larval stage, marked by focus on substrate consumption for larval growth, and the pupal stage, marked by differentiation of the BSF bacterial community composition [34], towards several bacterial taxa with diverse immune and defense functions [31]. For example, some *Providencia* spp., such as *P. rettgeri*, and members of the family *Bacillaceae* are known for their antimicrobial activity [72].

Transition into adults induced a complete turnover of the bacterial community composition, as illustrated by the relatively large distance between pupal and adult sampling points in Fig. 6B. At phylum level, *Pseudomonadota* dominated the adult stage, a shift from the previous dominance by *Actinomycetota* and *Bacillota*. Besides the large proportion of the “Minor zOTUs (< 5%)” category, indicating presence of many bacterial genera with low relative abundance, *Enterococcus* (zOTU 1, 4 and 17) was the only genus from the pupal stage that retained abundance over 5%. This is in contrast with another recent study [31], which reported overlap in bacterial communities between pupal and adult stages. Moreover, in our study, *Acetobacter* (zOTU 34, 15–16%) and *Ralstonia* (zOTU 19, 17–18%) emerged as dominant genera in the emerged flies at DAH 39. Members of *Acetobacter* sp. were previously reported in early adult stages, in which they were supposed to contribute to adult growth, especially when reared in poor nutrient conditions [73]. In contrast, *Ralstonia* spp. were not associated to the BSF bacterial community before.

Further development of the flies resulted in substantial discrepancies between the control and inoculated cycle. The bacterial community composition of flies from the control cycle further evolved from the composition observed at DAH 39, dominated by *Delftia* sp. (zOTU 24), *Enterococcus* sp. (zOTU 1, 4 and 17) and *Ralstonia* sp. (zOTU 19) by DAH 43 and 46. Conversely, flies from the inoculated cycle completely transformed their bacterial composition after DAH 39, with *Acinetobacter* (zOTU 25, 29, 33 and 42; 29–46%) as predominant genus. Indeed, *Acinetobacter* sp. was previously noted for its abundance in BSF adults, and members of this genus are suggested to play a role in nitrogen fixation to synthetise proteins, nucleotides or other biomolecules essential for growth and development of the insect [31]. At the final sampling day (DAH 46), *Morganella* (zOTU 27; 22%) emerged as another dominant genus, of which some members, for example *M. morganii*, are known for their antimicrobial activity against, for example, *E. coli* [72]. Despite substrate inoculation being the only variable factor between the control and inoculated cycle, the variability of the adults’ bacterial composition appeared implausible to be attributed to introduction of *E. coli* in the substrate. This assumption was supported by the PERMANOVA results, attributing only 9% of the variation between bacterial communities of the BSF life stages (Fig. 6B) to the inoculation of the substrate (R^2^ = 0.09, *p* < 0.001). In addition, culture-dependent experiments on vertical transmission stated that inoculated kanamycin-resistant *E. coli* was below the detection limit during the entire adult stage (Fig. 2B). Together with the consistency in bacterial composition until DAH 39, this strongly suggests that other environmental factors play a pivotal role in modulating the microbial community of BSF flies. Moreover, the high diversity and variation of the adult bacterial communities, characterised by numerous genera with low abundances and a prominent “Minor zOTUs” group (Fig. 5B), might be due to assembling their community from scratch. Moreover, microbial colonisation of the flies will occur immediately after emergence, as they start flying and having contact to surfaces, water and remaining puparia. Recent studies contradicting the assumption that BSF flies do not feed proposed that feed and water intake might partially reshape the flies’ microbiota [1, 33]. With only water intake and the presence of an inaccessible decaying substrate near the egg plates in this study, the few influencing factors might result in evolution of the bacterial community composition in different directions.

Furthermore, *Escherichia* sp. (zOTU 16) was detected in low abundances (< 3%) across all larval, prepupal and pupal samples, with no considerable increase observed in the inoculated cycle due to the same reasons as suggested for the substrate and frass. In fly samples, *Escherichia* sp. (zOTU 16) was only sporadically recovered with abundances below 4%. Consequently, it can be concluded that the introduction of *E. coli* in the substrate and BSF life stages did not have a significant impact on the bacterial community of the BSF.

In summary, it is clear that the bacterial community across BSF life stages is characterised by a dynamic nature, and is composed of both bacterial taxa acquired from the environment and others vertically transmitted across the life stages. Although inoculation of *E. coli* only minimally impacted the bacterial community composition of the BSF, future might investigate transmission of other microorganisms across more life cycles, also including the egg stage.

## Conclusions

This study underscored the dynamic character of the microbiota of consecutive BSF life stages and the associated substrate and frass. Observations for both the microbial counts and bacterial community composition emphasised relative consistency during the larval stage and first days of the prepupal stage. This microbial community composition was mainly shaped by the substrate composition. On the other hand, BSF larvae rearing resulted in a higher bacterial diversity of the frass. Although diversification of the bacterial community already occurred during the pupal stage, a complete shift in the bacterial community was observed during metamorphosis to the adult stage, accompanied by a strong reduction of all microbial counts, followed by revival of most general microbial groups.

Furthermore, vertical transmission of *E. coli* across BSF life stages was investigated through inoculation of approximately 7.0 log cfu/g of kanamycin-resistant *E. coli* in the BSF rearing substrate (DAH 8 and 18). Larvae rearing resulted in reduction of *E. coli* counts in the frass to below 5.0 log cfu/g. Although *E. coli* was taken up by the larvae, limited vertical transmission of *E. coli* was observed with a clear decreasing trend until the prepupal stage, while *E. coli* counts were below the detection limit of 1.0 log cfu/g for all BSF samples from the end of the pupal stage and the adult stage. Additionally, introduction of *E. coli* in the substrate did not have a substantial impact on the bacterial community composition of the substrate, frass or different BSF life stages. Consequently, it can be stated that using substates contaminated with *E. coli* for rearing BSF larvae does not necessarily pose a microbiological safety problem for the larvae. Nevertheless, vertical transmission of foodborne pathogens might be affected by several factors, such as the insect species, pathogen species and pathogen level, which leads to the fact that these findings can not be generalised. Hence, this study paves the way for further case studies on vertical transmission across consecutive life stages of BSF or other insect species, leading to more insight in pathogen transmission processes and microbiological food or feed safety associated with BSF or other insects.

### Electronic supplementary material

Below is the link to the electronic supplementary material.

**Additional file 1**: **Table S1**. Microbial counts of the substrate, frass and different BSF life stages, reared on the control substrate (chicken feed). Results are presented as the mean of 6 replicates (*n* = 3 cycle repetitions x 2 technical repetitions) ± standard deviation.

**Additional file 2**: **Table S2**. Bacterial diversity metrics (observed richness, Shannon diversity index and Simpson’s diversity index) of substrate and frass samples.

**Additional file 3**: **Table S3**. Bacterial diversity metrics (observed richness, Shannon diversity index and Simpson’s diversity index) of samples of different BSF life stages.

## Data Availability

The 16S rRNA gene sequencing dataset generated and analysed during the current study are available in the NCBI Sequence Read Archive under BioProject PRJNA1068952.

## Abbreviations

ASV: Amplicon sequence variants
a_w_: Water activity
BSF: Black soldier fly
DAH: Days after hatching
DRBC: Dichloran Rose Bengal Chloramphenicol agar
MRS: de Man, Rogosa and Sharpe agar
PCA: Plate Count Agar
PCoA: Principal Coordinates Analysis
PERMANOVA: Permutational multivariate analysis of variance
RH: Relative humidity
SRA: Sequence Read Archive
VRBG: Violet Red Bile Glucose agar
zOTU: Zero-radius operational taxonomic unit
