# Supplementary material for: Microbial dynamics and vertical transmission of Escherichia coli across consecutive life stages of the black soldier fly (Hermetia illucens)
Source: Anim Microbiome. 2024 May 26;6:29. doi: 10.1186/s42523-024-00317-4 (PMC11129375; doi:10.1186/s42523-024-00317-4)
Supplement: Supplementary file 1 — Additional file 1: Table S1. Microbial counts of the substrate, frass and different BSF life stages, reared on the control substrate (chicken feed). Results are presented as the mean of 6 replicates (n = 3 cycle repetitions x 2 technical repetitions) ± standard deviation. [file 42523_2024_317_MOESM1_ESM.docx]

**Table S1.** Microbial counts of the substrate, frass and different BSF life stages, reared on the control substrate (chicken feed). Results are presented as the mean of 6 replicates (n = 3 cycle repetitions x 2 technical repetitions) ± standard deviation.

|  |  | **Microbial counts (log cfu/g)** | | | | | | | | | | | | | | | | | |
| --- | --- | --- | --- | --- | --- | --- | --- | --- | --- | --- | --- | --- | --- | --- | --- | --- | --- | --- | --- |
| **Sample** | **Sampling time (DAH)** | **Total viable count** | | | **Enterobacteriaceae** | | | **Lactic acid bacteria** | | | **Aerobic endospores** | | | **Fungi** | | | ***E. coli* (kanamycin-resistant)** | | |
| Substrate | 8 | 8.4 | ± | 0.6^a^ | 8.2 | ± | 0.3^b^ | 8.2 | ± | 0.4^a^ | < 4.4 | ± | 1.0^a^ | 7.1 | ± | 0.1^a^ | < 1.5 | ± | 0.4^a^ |
| Substrate | 11 | 9.8 | ± | 0.4^b^ | 8.8 | ± | 0.5^b,c^ | 9.6 | ± | 0.6^b^ | 6.0 | ± | 1.5^a,b^ | 9.0 | ± | 0.1^b^ | < 1.4 | ± | 0.5^a^ |
| Substrate | 15 | 10.1 | ± | 0.7^b^ | 9.5 | ± | 0.5^c^ | 9.7 | ± | 0.7^b^ | 6.5 | ± | 1.6^b^ | 8.8 | ± | 0.3^b^ | < 1.3 | ± | 0.5^a^ |
| Frass | 18 | 9.3 | ± | 1.0^a,b^ | 8.2 | ± | 0.6^b^ | 8.9 | ± | 1.0^a,b^ | 5.8 | ± | 1.2^a,b^ | 7.1 | ± | 0.6^a^ | < 1.5 | ± | 0.4^a^ |
| Frass | 22 | 9.1 | ± | 1.2^a,b^ | 6.6 | ± | 0.6^a^ | 8.6 | ± | 0.7^a,b^ | 5.8 | ± | 1.1^a,b^ | 6.7 | ± | 0.4^a^ | < 1.1 | ± | 0.3^a^ |
| Larvae | 8 | 8.3 | ± | 0.7^c,d,e^ | 8.2 | ± | 0.4^c^ | 8.0 | ± | 0.6^d,e^ | < 4.8 | ± | 1.0^d^ | 7.6 | ± | 0.7^b,c^ | < 1.2 | ± | 0.4^a^ |
| Larvae | 11 | 8.9 | ± | 0.5^e,f^ | 8.3 | ± | 0.4^c^ | 8.8 | ± | 0.6^e^ | 4.9 | ± | 0.7^d^ | 8.5 | ± | 0.3^c^ | < 1.3 | ± | 0.5^a^ |
| Larvae | 15 | 9.0 | ± | 0.2^† f,g^ | 8.3 | ± | 0.5^† c^ | 8.7 | ± | 0.1^† e^ | 4.6 | ± | 0.6^† c,d^ | 8.5 | ± | 0.0^† c^ | < 1.0 | ± | 0.0^† a^ |
| Larvae | 18 | 8.6 | ± | 0.6^d,e,f^ | 8.1 | ± | 0.7^c^ | 8.0 | ± | 0.2^d^ | 5.4 | ± | 1.6^c,d^ | 7.7 | ± | 0.1^b,c^ | < 1.1 | ± | 0.1^a^ |
| Larvae | 22 | 8.3 | ± | 0.7^c,d,e,f^ | 8.0 | ± | 0.6^c^ | 7.6 | ± | 0.4^c,d^ | 5.1 | ± | 1.6^c,d^ | 6.3 | ± | 0.3^b^ | < 1.1 | ± | 0.2^a^ |
| Prepupae | 25 | 7.8 | ± | 0.4^c^ | 7.5 | ± | 0.5^b,c^ | 7.0 | ± | 0.2^b^ | 4.5 | ± | 2.2^b,c,d^ | 4.0 | ± | 1.4^a^ | < 1.0 | ± | 0.0^a^ |
| Prepupae | 29 | 7.8 | ± | 0.6^c,d^ | 7.3 | ± | 0.5^b,c^ | 6.7 | ± | 0.8^b,c^ | < 4.5 | ± | 0.6^c,d^ | < 4.0 | ± | 0.7^a^ | < 1.0 | ± | 0.0^a^ |
| Pupae | 32 | 7.9 | ± | 0.7^c,d^ | 7.4 | ± | 0.4^b,c^ | 7.0 | ± | 0.4^b,c^ | 4.2 | ± | 1.5^b,c,d^ | 3.8 | ± | 0.8^a^ | < 1.0 | ± | 0.0^a^ |
| Pupae | 36 | 7.7 | ± | 0.7^b,c^ | 7.1 | ± | 0.3^b,c^ | 6.6 | ± | 0.6^b^ | 5.0 | ± | 0.8^d^ | 4.0 | ± | 0.8^a^ | < 1.0 | ± | 0.0^a^ |
| Adult | 39 | 6.2 | ± | 0.9^a,b^ | < 3.6 | ± | 1.4^a^ | > 4.9 | ± | 0.7^a^ | < 3.1 | ± | 0.4^a,b^ | < 3.3 | ± | 1.0^a^ | < 1.0 | ± | 0.0^a^ |
| Adult | 43 | 6.2 | ± | 0.5^a^ | 6.2 | ± | 0.4^† b^ | 4.9 | ± | 0.6^a^ | 2.7 | ± | 0.2^a^ | < 3.6 | ± | 1.3^a^ | < 1.0 | ± | 0.0^a^ |
| Adult | 46 | 7.2 | ± | 1.3^a,c,g^ | 7.2 | ± | 1.1^† b,c^ | 5.4 | ± | 0.6^a^ | < 3.6 | ± | 0.8^b,c^ | < 3.7 | ± | 1.4^a^ | < 1.0 | ± | 0.0^a^ |

^†^ Results are the mean of 4 replicates (n = 4) ± standard deviation.

^a,b,c,d,e,f^ Means of samples of substrate/frass or life stages of BSF with the same letter in superscript in the same column do not differ significantly (p ≥ 0.05).
