# Supplementary material for: Microbial dynamics and vertical transmission of Escherichia coli across consecutive life stages of the black soldier fly (Hermetia illucens)
Source: Anim Microbiome. 2024 May 26;6:29. doi: 10.1186/s42523-024-00317-4 (PMC11129375; doi:10.1186/s42523-024-00317-4)
Supplement: Supplementary file 2 — Additional file 2: Table S2. Bacterial diversity metrics (observed richness, Shannon diversity index and Simpson’s diversity index) of substrate and frass samples. [file 42523_2024_317_MOESM2_ESM.docx]

**Table S2.** Bacterial diversity metrics (observed richness, Shannon diversity index and Simpson’s diversity index) of substrate and frass samples.

| **Sample name** | **Observed richness** | **Shannon diversity index** | **Simpson’s diversity index** |
| --- | --- | --- | --- |
| CF_SUB_8_1 | 42 | 2.779833 | 0.914199 |
| CF_SUB_8_2 | 43 | 2.690961 | 0.901783 |
| CF_SUB_11_1 | 45 | 2.806519 | 0.889137 |
| CF_SUB_11_2 | 49 | 2.830262 | 0.890723 |
| CF_SUB_15_1 | 54 | 2.908037 | 0.909183 |
| CF_SUB_15_2 | 53 | 2.971037 | 0.920866 |
| CF_FR_18_1 | 73 | 3.164759 | 0.923986 |
| CF_FR_18_2 | 79 | 3.184396 | 0.923514 |
| CF_FR_22_1 | 84 | 3.368122 | 0.94044 |
| CF_FR_22_2 | 87 | 3.410137 | 0.9439 |
| EC_SUB_8_1 | 41 | 2.724707 | 0.901571 |
| EC_SUB_8_2 | 42 | 2.673224 | 0.889333 |
| EC_SUB_8_3 | 43 | 2.706523 | 0.898583 |
| EC_SUB_11_1 | 44 | 2.739864 | 0.880203 |
| EC_SUB_11_2 | 49 | 2.752414 | 0.881706 |
| EC_SUB_11_3 | 49 | 2.777657 | 0.88809 |
| EC_SUB_15_1 | 58 | 3.04741 | 0.923508 |
| EC_SUB_15_2 | 53 | 3.09653 | 0.933103 |
| EC_SUB_15_3 | 56 | 3.091443 | 0.930271 |
| EC_FR_18_BEFORE_1 | 73 | 3.176642 | 0.928625 |
| EC_FR_18_BEFORE_2 | 63 | 3.098657 | 0.927422 |
| EC_FR_18_BEFORE_3 | 69 | 3.14846 | 0.929381 |
| EC_FR_18_AFTER_1 | 77 | 3.26859 | 0.932867 |
| EC_FR_18_AFTER_2 | 80 | 3.365477 | 0.940734 |
| EC_FR_18_AFTER_3 | 89 | 3.372692 | 0.93792 |
| EC_FR_22_1 | 95 | 3.517687 | 0.945011 |
| EC_FR_22_2 | 83 | 3.354701 | 0.938262 |
| EC_FR_22_3 | 87 | 3.389087 | 0.941223 |
