# Supplementary material for: Microbial dynamics and vertical transmission of Escherichia coli across consecutive life stages of the black soldier fly (Hermetia illucens)
Source: Anim Microbiome. 2024 May 26;6:29. doi: 10.1186/s42523-024-00317-4 (PMC11129375; doi:10.1186/s42523-024-00317-4)
Supplement: Supplementary file 3 — Additional file 3: Table S3. Bacterial diversity metrics (observed richness, Shannon diversity index and Simpson’s diversity index) of samples of different BSF life stages. [file 42523_2024_317_MOESM3_ESM.docx]

**Table S3.** Bacterial diversity metrics (observed richness, Shannon diversity index and Simpson’s diversity index) of samples of different BSF life stages.

| **Sample name** | **Observed richness** | **Shannon diversity index** | **Simpson’s diversity index** |
| --- | --- | --- | --- |
| CFL_8_1 | 51 | 2.686742 | 0.869946 |
| CFL_8_2 | 48 | 2.788778 | 0.892312 |
| CFL_11_1 | 45 | 2.46892 | 0.792906 |
| CFL_11_2 | 49 | 2.692926 | 0.851096 |
| CFL_15_1 | 45 | 2.222193 | 0.771814 |
| CFL_15_2 | 42 | 2.171233 | 0.766644 |
| CFL_18_1 | 39 | 2.311592 | 0.812458 |
| CFL_18_2 | 38 | 2.270089 | 0.812024 |
| CFPP_22_1 | 39 | 2.305897 | 0.822964 |
| CFPP_22_2 | 38 | 2.219069 | 0.800629 |
| CFPP_25_1 | 52 | 3.101332 | 0.936737 |
| CFPP_25_2 | 49 | 3.118545 | 0.938864 |
| CFP_29_1 | 53 | 3.155025 | 0.939097 |
| CFP_29_2 | 55 | 3.186139 | 0.940345 |
| CFP_32_1 | 61 | 3.286417 | 0.945176 |
| CFP_32_2 | 56 | 3.268725 | 0.946191 |
| CFFL_36_1 | 129 | 4.033812 | 0.966205 |
| CFFL_36_2 | 95 | 3.531651 | 0.940206 |
| CFFL_39_1 | 33 | 1.577314 | 0.524382 |
| CFFL_39_2 | 45 | 2.494782 | 0.846773 |
| CFFL_43_1 | 46 | 2.609089 | 0.868241 |
| CFFL_43_2 | 44 | 2.661239 | 0.887308 |
| ECL_8_1 | 42 | 2.314774 | 0.7845 |
| ECL_8_2 | 38 | 2.341804 | 0.802769 |
| ECL_8_3 | 37 | 2.360193 | 0.806401 |
| ECL_11_1 | 45 | 2.077753 | 0.760964 |
| ECL_11_2 | 47 | 2.119719 | 0.771623 |
| ECL_11_3 | 51 | 2.686742 | 0.869946 |
| ECL_15_1 | 48 | 2.788778 | 0.892312 |
| ECL_15_2 | 45 | 2.46892 | 0.792906 |

**Table S3.** Continued.

| **Sample name** | **Observed richness** | **Shannon diversity index** | **Simpson’s diversity index** |
| --- | --- | --- | --- |
| ECL_15_3 | 57 | 2.224318 | 0.788026 |
| ECL_18_BEFORE_1 | 52 | 2.357265 | 0.816364 |
| ECL_18_BEFORE _2 | 47 | 2.259613 | 0.808867 |
| ECL_18_BEFORE _3 | 38 | 1.948517 | 0.764256 |
| ECL_18_AFTER_1 | 46 | 2.276177 | 0.780611 |
| ECL_18_AFTER _2 | 45 | 2.298948 | 0.783046 |
| ECL_18_AFTER _3 | 46 | 2.341585 | 0.795393 |
| ECPP_22_1 | 44 | 2.648773 | 0.875384 |
| ECPP_22_2 | 41 | 2.498427 | 0.855091 |
| ECPP_22_3 | 41 | 2.65693 | 0.875776 |
| ECPP_25_1 | 63 | 3.284234 | 0.945647 |
| ECPP_25_2 | 66 | 3.356134 | 0.948562 |
| ECPP_25_3 | 122 | 4.012352 | 0.969437 |
| ECP_29_1 | 75 | 3.396221 | 0.945077 |
| ECP_29_2 | 61 | 3.24635 | 0.938678 |
| ECP_29_3 | 75 | 3.297247 | 0.938936 |
| ECP_32_1 | 65 | 3.287754 | 0.937159 |
| ECP_32_2 | 70 | 3.443034 | 0.948584 |
| ECP_32_3 | 100 | 3.525538 | 0.933312 |
| ECFL_36_1 | 118 | 4.033191 | 0.967739 |
| ECFL_36_2 | 47 | 2.498326 | 0.844861 |
| ECFL_36_3 | 39 | 2.245564 | 0.819608 |
| ECFL_39_1 | 151 | 4.347308 | 0.976599 |
| ECFL_39_2 | 57 | 2.735463 | 0.862607 |
| ECFL_39_3 | 78 | 3.463936 | 0.947855 |
| ECFL_43_1 | 55 | 2.609677 | 0.836203 |
| ECFL_43_2 | 26 | 2.201593 | 0.837227 |
| ECFL_43_3 | 29 | 0.928583 | 0.333933 |
